# Hyponatraemia Induced by Terlipressin in Patients Diagnosed with Decompensated Liver Cirrhosis and Acute Variceal Bleeding

**DOI:** 10.3390/medicines12020007

**Published:** 2025-03-28

**Authors:** Mahmoud Elshehawy, Richel Merin Panicker, Alaa Amr Abdelgawad, Patrick Anthony Ball, Hana Morrissey

**Affiliations:** 1 West Midlands Deanery, Birmingham B2 4HQ, UK; mahmoud.elshehawy@nhs.net; 2 General Internal Medicine, Royal Shrewsbury Hospital, Shrewsbury SY3 8XQ, UK; richel.panicker@nhs.net; 3 Mansoura University Hospitals, Mansoura 35516, Egypt; alaaamrjawad1@gmail.com; 4 School of Medical and Dental Sciences, Charles Sturt University, Bathurst, NSW 2795, Australia; pba87218@gmail.com; 5 Independent Researcher, 6036 Perth, Australia; 6 School of Pharmacy, University of Ruhuna, Matara 81000, Sri Lanka

**Keywords:** terlipressin, hyponatraemia, liver cirrhosis, adverse drug reaction, fluid retention, variceal bleeding

## Abstract

**Background:** Hyponatraemia is a rare but potentially life-threatening complication of terlipressin therapy. **Case history:** In the current case, a 39-year-old female with decompensated liver cirrhosis (Child-Pugh C) and acute variceal bleeding experienced a precipitous decline in serum sodium—from 136 mmol/L to 115 mmol/L—within 48 h of initiating terlipressin therapy. This was accompanied by marked fluid retention, reduced urine output, and symptoms of confusion and agitation. Laboratory tests confirmed dilutional hyponatraemia, characterized by urinary sodium <20 mmol/L and urine osmolality <100 mOsm/kg, indicating excessive free water reabsorption. **Outcomes:** The prompt discontinuation of terlipressin, fluid restriction and the cautious administration of hypertonic sodium chloride solution (2.7% NaCl) achieved a gradual normalization of sodium levels and resolution of symptoms. Fluid balance monitoring revealed a marked diuretic response following terlipressin cessation. This case aligns with existing reports, emphasizing the dual vasopressin receptor activity of terlipressin and its capacity to induce hyponatraemia, particularly in cirrhotic patients with preserved renal function and higher baseline sodium levels. **Conclusions:** This case and a literature review underscored the critical need for early fluid balance monitoring to detect retention. This case highlights the importance of individualized risk assessment, multidisciplinary management, and vigilant sodium correction to avoid complications. Practical recommendations are outlined to aid clinicians in the recognition and management of terlipressin-induced hyponatraemia.

## 1. Background

Terlipressin is a synthetic vasopressin analogue, with a higher affinity to vasopressin-1 (V1) receptors in the gastroesophageal tract than for vasopressin-2 (V2) receptors in the kidneys. The effect on V1 receptors is therapeutically desirable (management of gastroesophageal haemorrhage and hepatorenal syndrome), while the effect on the V2 receptors is rare, unwanted (dilutional hyponatraemia) and under-recognised [1].

The incidence of terlipressin-induced hyponatraemia ranges from 0.1% in controlled trials to 6% in real-world applications. Several risk factors predispose patients to this complication, including higher baseline sodium levels, younger age and preserved hepatic and renal function [2]. Mechanistically, V2 receptor activation facilitates the insertion of aquaporin channels in renal tubules, promoting free water retention, plasma sodium dilution, and, in susceptible individuals, symptomatic hyponatraemia. This process is often accompanied by significant fluid retention, as observed in published cases and this report [3].

## 2. Aim

By sharing this case, we aim to improve clinician awareness and contribute to the development of standardized protocols for monitoring and managing terlipressin-induced adverse effects, ensuring safer and more effective therapeutic outcomes.

## 3. Methods and Design

### 3.1. Rationale and Objectives

A comprehensive search of the literature was conducted on 16 October 2024 through the Shrewsbury and Telford Health Libraries (Medline and EMBASE), United Kingdom. After title and duplication review, 37 original research articles were identified over 6 search episodes (Table 1); after abstract review, only 16 articles were found to be relevant to this case. The only limitation of this search was the use of the English language and the exclusion of articles we could not source in the English language after contacting the publishing journal and the corresponding authors.

The literature review underscores the rarity of this complication [4] and highlights a critical gap in identifying reliable predictive markers for terlipressin-induced hyponatraemia [2,5,6,7,8,9,10]. Importantly, fluid retention frequently precedes sodium derangements by approximately 24 h, making early fluid balance monitoring an essential, non-invasive strategy for detecting at-risk patients [4,11]. This observation, reaffirmed in our patient, highlights the potential for timely intervention to prevent progression to severe hyponatraemia and its associated complications. This case seeks to advance the clinical understanding of terlipressin-induced hyponatraemia by synthesizing evidence from similar reports and offering practical recommendations for prevention and management.

### 3.2. Case Presentation and Clinical Course

A 39-year-old female with a complex medical history of decompensated alcoholic liver cirrhosis (Child-Pugh C), oesophageal varices, portal hypertension, and recurrent hepatic encephalopathy with no history of previous episodes of hyponatraemia presented to the emergency department following three episodes of massive hematemesis.

On arrival, she was hypotensive (blood pressure: 80/50 mmHg) and tachycardic (heart rate: 96 bpm), requiring immediate resuscitation with intravenous crystalloids, blood products and vasopressors. Urgent endoscopy revealed actively bleeding oesophageal varices, successfully controlled using endoscopic band ligation. The patient had been taking 20 mg citalopram once daily (hyponatraemia is a possible side effect) and using a salbutamol inhaler (hypokalaemia is possible side effect) as required for many years; both were suspended during the hospital admission. Omeprazole 40 mg twice daily intravenously was also commenced.

Given the severity of her bleeding, terlipressin was initiated at 1 mg every 4 h following haemostasis to reduce the risk of rebleeding. Baseline laboratory results are shown in Table 2.

Within 48 h of terlipressin initiation, the patient developed severe symptomatic hyponatraemia, with serum sodium levels falling to 115 mmol/L. She exhibited confusion, agitation, nausea, and generalized weakness. Fluid balance monitoring revealed significant fluid retention (fluid intake of 3000 mL/day and urine output of 1200–1500 mL/day). Laboratory investigations confirmed dilutional hyponatraemia (urinary sodium: <20 mmol/L and urine osmolality: <100 mOsm/kg).

Measured on the WHO-UMC system [12], the adverse event can be classified as possible/likely (event or laboratory test abnormality, with reasonable time relationship to drug intake; could also be explained by disease or other drugs, unlikely to be attributed to disease or other drugs, response to withdrawal clinically reasonable and rechallenge not required). This classification was made after excluding citalopram, infection stress and omeprazole as the cause or contributing cause for the hyponatraemia.

### 3.3. Differential Diagnosis and Key Investigations

Given the complexity of the patient’s condition, alternative causes of hyponatraemia were systematically evaluated and excluded as follows:

1. Hepatorenal syndrome: Excluded due to the absence of renal impairment, as evidenced by preserved renal function (serum creatinine of 78 µmol/L, stable eGFR). This is inconsistent with the hallmark findings of hepatorenal syndrome, which include oliguria and progressive renal dysfunction [13].
2. Adrenal Insufficiency: Ruled out based on normal random cortisol levels and the absence of associated clinical features, such as hyperkalaemia, hypotension, or fatigue. These findings did not support adrenal dysfunction as a contributing factor.
3. Syndrome of Inappropriate Antidiuretic Hormone (SIADH): Deemed highly unlikely, as the observed hyponatraemia was dilutional in nature rather than SIADH-mediated water imbalance [14].
4. Excessive diuretic use: Ruled out as the patient was not prescribed a loop or thiazide diuretic, which are common contributors to diuretic-induced hyponatraemia.
5. V2 receptor activation secondary to terlipressin: Urinary sodium of <20 mmol/L, urine osmolality of <100 mOsm/kg, and then a rapid improvement in serum sodium levels and resolution of symptoms upon discontinuation of terlipressin [2].

### 3.4. Clinical Rationale for Diagnosis

The activation of V2 receptors in the renal collecting ducts by terlipressin is further exacerbated in patients with cirrhosis due to existing fluid imbalances and altered renal handling of electrolytes [15].

In this case, the patient exhibited significant fluid retention during terlipressin therapy, with a daily fluid intake exceeding 3000 mL and a reduced urine output of 1200–1500 mL/day. Following the discontinuation of terlipressin, a rapid diuresis of 8000 mL/day was observed, with normalization of serum sodium and the resolution of symptoms.

These findings suggest excessive free water reabsorption due to vasopressin V2 receptor activation by terlipressin. The clinical sequence strongly suggested a causal relationship between terlipressin therapy and the rapid development of hyponatraemia, particularly given the observed fluid retention preceding the sodium decline [16]. These findings are consistent with published reports describing similar outcomes in cirrhotic patients receiving terlipressin [6,7,17]. The discontinuation of terlipressin led to a positive reversibility response, marked by the progressive normalization of sodium levels and the resolution of symptoms, further supporting the link between the drug and this adverse event [12].

### 3.5. Treatment

Sodium levels started to slowly drop from 136 mmol/L on admission to 112 mmol/L on day 3 of admission, in the early morning. Terlipressin was promptly discontinued (in response to the rapid onset of severe hyponatraemia from 136 on admission to 115 mmol/L with significant fluid retention), and the bleeding was controlled.

The management plan focused on daily monitoring and restoring sodium balance and mitigating further complications through a combination of fluid restriction (1000 mL/day) and the cautious administration of hypertonic sodium chloride solution (2.7% NaCl). Additionally, intravenous furosemide was carefully introduced to support diuresis and manage the fluid overload. Within 72 h, the patient’s serum sodium levels improved to 133 mmol/L, and her symptoms (including confusion, agitation, and generalized weakness) completely resolved. Fluid restriction was maintained at 1250 mL and a positive fluid balance was monitored and improved from +465 (Na 112 mmol/L) on day 3 to +68 on day 5 (Na 116 mmol/L); sodium levels improved after that, to 133 mmol/L on day 6 and 140 mmol/L on day 7.

The decision to discontinue terlipressin was based on its well-documented association with dilutional hyponatraemia, driven by vasopressin V2 receptor activation in the renal collecting ducts. The concurrent use of hypertonic sodium chloride solution aligned with clinical guidelines for managing acute sodium derangements in the setting of advanced liver disease [18]. The patient’s positive response to these interventions, marked by rapid clinical improvement and normalization of sodium levels, strongly supported the causal role of terlipressin.

Throughout this period, strict fluid balance monitoring and serial measurements of serum sodium ensured safe correction while minimizing the risk of complications such as osmotic demyelination syndrome [16]. This multidisciplinary approach highlights the importance of early recognition and prompt intervention in managing terlipressin-induced adverse effects.

### 3.6. Outcome

The patient was discharged on day 7 post-admission with a normal serum sodium level of 136 mmol/L (consistent with her baseline). At discharge, the patient demonstrated a complete resolution of all presenting hyponatraemia symptoms, including confusion, agitation, and generalized weakness. A detailed discharge plan was implemented, emphasizing the importance of long-term fluid balance monitoring and awareness of early signs of hyponatraemia. She was advised to maintain a tailored fluid intake to minimize the risk of recurrence. The patient was scheduled for a structured outpatient follow-up with the hepatology clinic to monitor for signs of fluid imbalance, assess hepatic function, and support continued recovery. Additionally, a multidisciplinary care approach involving hepatology and nephrology specialists was established to provide comprehensive oversight of her complex medical needs. This collaboration aimed to optimize long-term outcomes by addressing both liver disease and potential renal implications, ensuring a holistic and patient-centred management strategy.

### 3.7. Follow-Up and Monitoring

A tailored follow-up plan was implemented, focusing on the following:

1. Regular monitoring: Weekly serum sodium evaluations during the initial recovery phase, transitioning to biweekly assessments over three months. This strategy aimed to ensure sustained electrolyte balance and detect early signs of recurrence.
2. Fluid management education: The patient was educated on maintaining a balanced fluid intake, tailored to their clinical condition. Specific advice included recognizing signs of fluid retention and seeking medical attention promptly if symptoms arose.
3. Multidisciplinary reviews: Regular hepatology and nephrology consultations were arranged to monitor liver and renal function, evaluate fluid retention risks, and address any complications, ensuring a holistic and patient-centred approach to care.

At the 4-week follow-up, the patient remained asymptomatic, with stable sodium levels and no recurrence of hyponatraemia. These findings confirm the effectiveness of the management strategy and highlight the critical role of multidisciplinary and individualized care.

## 4. Discussion

Hyponatraemia has been reported as rare, with an incidence of 0.1% to 6%. A 2020 retrospective review of 219 cases [17] found a reduction in serum sodium in 62.6% of cases and severe hyponatraemia in 33.3%, whilst a series of 674 patients [7] reported sodium reduction in 26.3% and severe hyponatraemia in 13%. Conversely, a systematic review and meta-analysis covering 7257 cases [4] reported the pooled incidence for all serious adverse events of 5%, and pooled incidence of hyponatraemia of 9%.

In this case, terlipressin was administered by bolus injection every 4 hours. The review concluded that the duration of action is less than 4 h, and that continuous infusion is the preferred method of administration.

This case closely mirrors findings from previously published reports on terlipressin-induced hyponatraemia, with the activation of vasopressin V2 receptors in the renal collecting ducts identified as a pivotal pathophysiological mechanism [17,19]. The resulting excessive water reabsorption and dilutional hyponatraemia have been consistently reported in cirrhotic patients treated with terlipressin [11]. Importantly, this case reinforces that significant fluid retention within the first 24 h of therapy serves as a critical early warning sign for impending hyponatraemia, and a negative correlation was found with renal function [17], suggesting that impaired sodium metabolism in the kidney affects the ability to retain water. The observations in this case align with the growing evidence supporting the need for early fluid balance monitoring as an effective and non-invasive strategy to identify patients at risk [11,15]. Proactive interventions, including timely fluid restriction and sodium correction, have been shown to mitigate severe complications [20]. This approach is particularly crucial in high-risk populations, such as those with pre-existing cirrhosis, preserved renal function, and higher baseline sodium levels, who are more susceptible to this adverse effect [11].

This case underscores the delicate therapeutic balance required in managing acute variceal bleeding with terlipressin. While the drug remains indispensable in controlling haemorrhage, its potential to cause significant adverse effects, such as hyponatraemia, necessitates vigilant monitoring and individualized treatment plans. Patients with baseline fluid imbalances, ascites, or renal susceptibility may benefit from tailored dosing and closer observation to optimize outcomes while minimizing risks.

The heterogeneity found by Shang et al. [4], which was not resolved by meta-regression analyses, suggests that there is still more to learn in optimizing management of this adverse effect. By synthesizing evidence from similar reports, this case highlights actionable insights that can guide clinicians in recognizing early indicators of terlipressin-induced complications and implementing preventive strategies. It further contributes to the evolving discourse on balancing the benefits and risks of terlipressin therapy in high-risk patient populations.

Terlipressin-induced hyponatraemia, while recognized, remains a rare and underreported complication, posing diagnostic and management challenges, especially in patients with complex comorbidities like cirrhosis. This case highlights the interplay of multiple risk factors and emphasizes the importance of individualized therapeutic strategies and vigilant monitoring. It contributes to the growing body of evidence on this critical but often underappreciated adverse effect of terlipressin therapy.

This effect was amplified in the present case due to several baseline risk factors:

1. Cirrhosis-associated fluid dysregulation: chronic liver dysfunction disrupted the patient’s ability to maintain fluid homeostasis, increasing vulnerability to water retention [4].
2. Compromised renal reserve: although baseline renal function was preserved, subtle renal insufficiency in the setting of advanced cirrhosis likely predisposed the patient to electrolyte disturbances [11,15].
3. Concurrent volume resuscitation: the use of intravenous fluids further exacerbated fluid shifts, compounding the dilutional effects of terlipressin-induced water retention.

These factors align with prior studies [2], which identified fluid retention within the first 24 h of therapy as a strong precursor to hyponatraemia. The rapid onset of symptoms and laboratory findings in this case underscore the importance of recognizing these risk factors early.

This case mirrors findings from previously reported instances of terlipressin-induced hyponatraemia. The observed positive dechallenge–rechallenge pattern of rapid symptom onset during therapy and resolution upon discontinuation corroborates the causal relationship. Similar cases described by Šíma et al. [2] and Kang et al. [10] emphasize the role of baseline sodium levels, hepatic reserve, and early fluid retention as predictors of this adverse effect.

While milder cases may resolve spontaneously, this patient required active therapeutic intervention, highlighting the variability in clinical presentations, the potential seriousness of this complication and the need for further case reports to identify optimal management strategies.

### 4.1. Learning Points

1. Prompt recognition of terlipressin-induced hyponatraemia and immediate discontinuation of the drug are essential to prevent complications.
2. Fluid restriction, careful administration of hypertonic saline and diuretic support are effective in resolving sodium imbalance and fluid overload.
3. Ongoing monitoring, patient education and coordinated specialist input are integral to optimizing outcomes, particularly in complex cases involving cirrhosis.
4. This case provides clear evidence of terlipressin-induced hyponatraemia (within 32 h of initiation of therapy) and reversibility response on discontinuation.
5. The findings support terlipressin vasopressin V2 receptor activation as a mechanism driving renal water retention and sodium dilution.
6. Risk stratification of patients with advanced liver disease, renal dysfunction and baseline sodium levels emerged as key risk factors for hyponatraemia outcomes.

### 4.2. Clinical Relevance and Future Directions

1. This case aligns with the existing literature, while introducing new insights into monitoring and management strategies for terlipressin-induced hyponatraemia.
2. By sharing this case, we aim to increase awareness among clinicians and promote the development of standardized protocols to enhance the safety and efficacy of terlipressin therapy.
3. Clinicians must carefully weigh the life-saving benefits of terlipressin in managing variceal bleeding against the potential risks, particularly in patients predisposed to fluid imbalances due to cirrhosis or renal dysfunction.
4. Future studies should focus on identifying early predictive markers of hyponatraemia and validating routine monitoring protocols that incorporate fluid balance assessments.
5. Patients receiving terlipressin should be educated about the importance of fluid balance and taught to recognize early symptoms of complications, such as confusion or fatigue.
6. Close clinical follow-up remains essential to prevent recurrence and ensure long-term safety.

## 5. Conclusions

Terlipressin is a cornerstone therapy in managing acute variceal bleeding, with its efficacy well-documented in reducing portal pressure and controlling haemorrhage in patients with cirrhosis and portal hypertension. This case highlights terlipressin-induced hyponatraemia as a rare yet clinically significant complication. Insights into its pathophysiology, risk factors and management provide valuable guidance for clinicians. Early recognition, prompt discontinuation of terlipressin and multidisciplinary collaboration were pivotal in ensuring a successful outcome. As the use of terlipressin continues to expand, clinicians must remain vigilant and implement evidence-based monitoring strategies to optimize patient care. Such complications pose diagnostic and management challenges, particularly in high-risk patients, including those with cirrhosis, renal impairment and other comorbidities. This case emphasizes the need for vigilance, individualized care and proactive therapeutic strategies in addressing such adverse effects.

## Figures and Tables

**Table 1:** Search strategy.

Search (S) Number	Query	Limiters/Expanders	Last Run Via	Results
S6	S1 AND S5	Expanders—Apply equivalent subjects Search modes—Find all my search terms	Interface—EBSCOhost Research Databases Search Screen—Advanced Search Database—MEDLINE	50
S5	S2 OR S3 OR S4	Expanders—Apply equivalent subjects Search modes—Find all my search terms	Interface—EBSCOhost Research Databases Search Screen—Advanced Search Database—MEDLINE	1283
S4	“terlipressin acetate”	Expanders—Apply equivalent subjects Search modes—Find all my search terms	Interface—EBSCOhost Research Databases Search Screen—Advanced Search Database—MEDLINE	3
S3	“glypressin”	Expanders—Apply equivalent subjects Search modes—Find all my search terms	Interface—EBSCOhost Research Databases Search Screen—Advanced Search Database—MEDLINE	168
S2	(MH “Terlipressin”) OR “terlipressin”	Expanders—Apply equivalent subjects Search modes—Find all my search terms	Interface—EBSCOhost Research Databases Search Screen—Advanced Search Database—MEDLINE	1256
S1	(MH “Hyponatremia”) OR “hyponatremia”	Expanders—Apply equivalent subjects Search modes—Find all my search terms	Interface—EBSCOhost Research Databases Search Screen—Advanced Search Database—MEDLINE	16,732

**Table 2:** Baseline laboratory results.

Test	Results	Interpretation
Serum sodium	136 mmol/L (135–145 mmol/L)	Normal
Serum creatinine	78 µmol/L (45–90 µmol/L)	Normal
Serum potassium	4.2 mmol/L (3.5–5.2 mmol/L)	Normal
Serum albumin	28 g/L (60–80 g/L)	Hypoalbuminemia
Total bilirubin	57 µmol/L (<20 µmol/L)	Hyperbilirubinemia
INR (International normalised ratio)	2.3	Normal

## Data Availability

All available data was included in this published report.
